# Estimating the Aboveground Carbon Density of Coniferous Forests by Combining Airborne LiDAR and Allometry Models at Plot Level

**DOI:** 10.3389/fpls.2019.00917

**Published:** 2019-07-10

**Authors:** Hongke Hao, Weizhong Li, Xuan Zhao, Qingrui Chang, Pengxiang Zhao

**Affiliations:** ^1^College of Natural Resources and Environment, Northwest A&F University, Yangling, China; ^2^College of Forestry, Northwest A&F University, Yangling, China; ^3^College of Landscape Architecture and Arts, Northwest A&F University, Yangling, China

**Keywords:** LiDAR, AGB, ACD, allometry model, NPC, CHM, TCH

## Abstract

Forest carbon density is an important indicator for evaluating forest carbon sink capacities. Accurate carbon density estimation is the basis for studying the response mechanisms of forest ecosystems to global climate change. Airborne light detection and ranging (LiDAR) technology can acquire the vertical structure parameters of forests with a higher precision and penetration ability than traditional optical remote sensing. Combining top of canopy height model (TCH) and allometry models, this paper constructed two prediction models of aboveground carbon density (ACD) with 94 square plots in northwestern China: one model is plot-averaged height-based power model and the other is plot-averaged daisy-chain model. The correlation coefficients (*R*^2^) were 0.6725 and 0.6761, which are significantly higher than the correlation coefficients of the traditional percentile model (*R*^2^ = 0.5910). In addition, the correlation between TCH and ACD was significantly better than that between plot-averaged height (AvgH) and ACD, and Lorey’s height (LorH) had no significant correlation with ACD. We also found that plot-level basal area (BA) was a dominant factor in ACD prediction, with a correlation coefficient reaching 0.9182, but this subject requires field investigation. The two models proposed in this study provide a simple and easy approach for estimating ACD in coniferous forests, which can replace the traditional LiDAR percentile method completely.

## Introduction

Forest carbon storage accounts for 82.5% of terrestrial vegetation carbon storage, which is the main component of the vegetation carbon sink (Cusack et al., 2014; Kauranne et al., 2017). Accurate calculations of forest carbon stocks are a hot topic in the field of forest carbon sink research. At present, large-scale estimations of forest carbon sinks are mainly realized by means of traditional optical remote sensing. Generally, the relationship between field survey data and remote sensing extraction parameters is established first and then extrapolated to the whole research scope; this technique is essentially remote sensing-assisted sampling surveying (Drake et al., 2003; Chi et al., 2017; Koju et al., 2018).

Traditional optical remote sensing can extract the spectral information and horizontal structure information of vegetation. However, with increasing biomass, saturation occurs easily, which affects the estimation accuracy of forest carbon storage (Zhao et al., 2016). Light detection and ranging (LiDAR) detects the distance between a sensor and target by emitting laser pulses and receiving reflections from the ground object. Thus, LiDAR can acquire high-precision three-dimensional information of the object. Furthermore, LiDAR has a certain penetrating ability and can obtain vertical structure information of forests, improving the estimation accuracy of forest height and structure and forest carbon storage (Dubayah and Drake, 2000; Naesset and Bjerknes, 2001; Hudak et al., 2002; Gwenzi and Lefsky, 2014).

With the big-data progress and increasing storage space in recent years, airborne LiDAR has become an important means of forest resource surveys and carbon storage research (Guo et al., 2017; Swetnam et al., 2017). Data processing methods are mainly divided into plot-based inversion and individual tree-based inversion. However, due to the large number of trees, complex spatial structure of forests and canopy shielding effect, single-tree segmentation algorithms are not yet mature. Therefore, developing plot-based inversion methods is indispensable (Ayrey et al., 2017; Dechesne et al., 2017).

There are two main approaches for the estimation of carbon density based on plots. One approach is the use of a variety of machine learning algorithms to establish the relationship between measured carbon density and LiDAR percentile metrics, which can make full use of the information contained in the point cloud to obtain increasing precision (Zhao et al., 2011; McRoberts et al., 2016). However, the modeling process is a black box operation, and the prediction results are difficult to explain. The other approach is the establishment of LiDAR inversion models directly based on allometry models (Mascaro et al., 2011; Asner and Mascaro, 2014). The premise of this method is that there is a similar allometric growth law for plot-level biomass and single-tree biomass. The key to this approach is finding the appropriate allometric growth model and the corresponding LiDAR extraction parameters.

A multiple linear regression model based on LiDAR percentiles is a popular method for estimating forest carbon density or biomass, which is widely used and has acceptable precision in different forest area (e.g., Boudreau et al., 2008; Zhao et al., 2009; Ferraz et al., 2016; Jimenez-Berni et al., 2018). Among them, Naesset and Gobakken (2008) explained 88% variation in aboveground biomass (AGB) and 85% belowground biomass using LiDAR derived variables in boreal forest (1395 circular sample plots with size 200–400 m^2^); Levick et al. (2016) used similar methods to obtain the fitting accuracy of 92% in 1 ha plots and 68% in 0.05 ha plots in temperate forest; Cao et al. (2016) established two regression models for estimating the AGB using multi-temporal LiDAR data of subtropical forest with *R*^2^ of 0.74 and 0.79 in 0.09 ha plots, respectively; Dubayah et al. (2010) estimated AGB in tropical forest with an *R*^2^ of 0.90 in 0.5 ha plots. Previous studies have demonstrated that the accuracy and form of percentiles models are closely related to the LiDAR instruments (Naesset, 2009; Silva et al., 2017) and plot size (Maltamo et al., 2011; Mascaro et al., 2011) except for intrinsic characteristics of forest.

In this study, we attempt to find a simple plot-based LiDAR extraction parameter, establish allometry models of the aboveground carbon density (ACD) of the northern coniferous forest, and evaluate the accuracy of these models. The objectives of this study are (1) the selection of the best parameter for ACD prediction from the following three plot-based LiDAR extraction parameters: top of canopy height (TCH), AVG (plot-averaged height), and Lorey’s height (LorH); (2) the proposal of direct and indirect fitting models of TCH and ACD and comparison of their accuracy and (3) calculation of the ACD of the study area with the proposed models and comparisons of the results and spatial distribution characteristics.

## Materials and Methods

### Study Area

**FIGURE 1:**
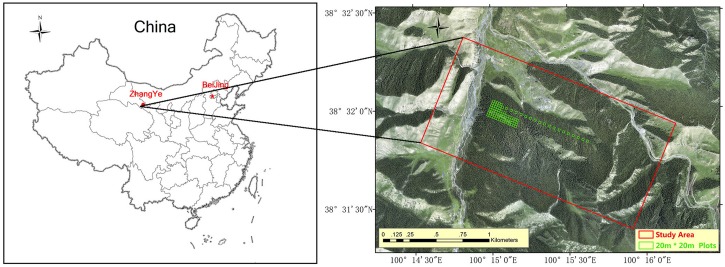
Study area and 94 plots.

The study was conducted 50 km southwest of Zhangye City, Gansu Province, Northwest China (Figure 1). The study area is approximately 264 ha, and its centre is at 100°15′E, 38°32′N. The elevation ranges from 2700 to 3200 m, the annual rainfall is 200 to 500 mm, and the monthly average temperature is 5.4 to 19.6°C. The main vegetation is natural pure Qinghai spruce (*Picea crassifolia*) forest, which has both naturally renewed young trees and tall over-ripe old trees. The ecoregion classification is “cascade pure conifer forest.”

### LiDAR Data and Processing

The LiDAR data used in this study were acquired on June 2008 using a LiteMapper 5600 instrument that recorded up to five returns per pulse, along with their intensity. The average flight altitude was 3560 m, the relative height over ground was 760 m, and the flight speed was 227 km/h. The laser scanner adopted RIEGL LMS-Q560, and the wavelength was 1550 nm. The laser pulse width was 3.5 ns, and the laser pulse divergence angle was less than or equal to 0.5 mrad. The LiDAR point cloud used the WGS84 coordinate system and the UTM projection zone 47 in the northern hemisphere. To increase the point density, the flight was repeated seven times over the study area with a side overlap of approximately 90%. As a result, the average point cloud interval was decreased to 0.54 m, and the average point cloud density was 3.43/m^2^.

Subsequently, a set of metrics (Table 1) was derived from the point cloud using the LAStools software package^1^. The main processing steps were as follows: (1) the point cloud was filtered and classified to ground, trees and noise; (2) the normalized point cloud (NPC, also referred to as height above ground) was calculated; (3) height percentiles, density percentiles and canopy cover (CC) were derived from the NPC corresponding to each plot; (4) the digital surface model (DSM) and digital elevation model (DEM)were interpolated from the first echo and the last echo of the point cloud, respectively. The canopy height model (CHM) was the difference of the first two. (5) The TCH was extracted from the CHM based on each plot (the mean value of 400 pixels per plot).

**TABLE 1:** Metrics derived from LiDAR and field investigation data.

**LiDAR Metric**	**Description**	**Origin Source**
TCH	Top of canopy height of plot	CHM (canopy height model)
h25…h95	Height percentiles	NPC (normalized point cloud)
d25…d95	Density percentiles	
CC	Canopy closure	
AvgH	Average height of plot	Field investigation
LorH	Lorey’s height of plot	
BA	Base area of plot	

In addition, in order to explore the effect of CHM pixel size on ACD prediction, we generated 10 CHMs from NPC, with pixel sizes from 1 to 10 m. When the pixel location of CHM corresponds to a laser point, the point’s height value is used as the pixel value. If the location corresponds to multiple laser points, the average value of height is used as the pixel value. For the pixels without corresponding laser point, inverse distance weighted (IDW) is adopted for interpolation, which can ensure smooth transition between the target pixel and surrounding pixels (Montealegre et al., 2015).

### Field Data and Processing

To calibrate and validate the models, the plot data were acquired simultaneously with the LiDAR data. A total of 94 square plots (20 m × 20 m), which included 5734 trees, were used. The four corners and the centre of each plot were measured using differential GPS (DGPS), and the error was less than 10 cm. For each tree with a diameter at breast height (DBH) greater than 5 cm, the tree type, diameter, height to crown base, crown width in cardinal directions, crown class, and crown transparency were measured. DBH was measured on all trees using a diameter tape, and the heights of all trees were measured using a laser ranging hypsometer with theoretical accuracy up to the decimeter level. Considering the canopy occlusion and human error, the average accuracy of the measured tree height was better than 0.5 m.

Using the species-specific allometry Eqs 1–4 in the study area (Wang et al., 1998; He et al., 2013), tree biomass components (stem, branch, foliage and fruit) were calculated from DBH and height. The AGB of each tree was equal to the sum of the AGB components and was then summed to obtain the AGB of each plot. These equations were constructed by destructive sampling and the fitting precision reached 0.9887, 0.9568, 0.8662, and 0.9340, respectively. Because the study area is a nature reserve and pure spruce forest with little human disturbance, it is believed that the field-estimated AGB of this study can also achieve such accuracy. Finally, the AGB was converted to the ACD using the conversion coefficient of 0.5034, which was attained using the potassium dichromate oxidation method on samples by Wang et al. (2000) in the same area. Additionally, Lorey’s height (LorH; Table 1) was also calculated based on each plot using Eq. 5, and the LorH values were compared with the TCH values extracted from LiDAR. For the same purpose, the average height (AvgH) of the plots was also calculated.

(1) $$\mathrm{Biomass}\,\_\,\mathrm{stem}=0.0478\times {\left(D^{2}\times H\right)}^{0.8665}$$

(2) $$\mathrm{Biomass}\,\_\,\mathrm{branch}=0.0061\times {\left(D^{2}\times H\right)}^{0.8905}$$

(3) $$\mathrm{Biomass}\,\_\,\mathrm{foliage}=0.2650\times {\left(D^{2}\times H\right)}^{0.4701}$$

(4) $$\mathrm{Biomass}\,\_\,\mathrm{fruit}=0.0342\times {\left(D^{2}\times H\right)}^{0.5779}$$

(5) $$\mathrm{LorH}=\sum {\mathrm{BA}}_{i}\,H_{i}/\sum {\mathrm{BA}}_{i}$$

where *D* is DBH, *H* is tree height, BA*_*i*_* and *H*_*i*_ are the basal area and the height of the *i*th tree, respectively, and *a* and *b* are regression fitting coefficients.

### Plot Allometry Models

The use of an allometry model is the main means of forest biomass calculation. This type of model is obtained by the regression of the sample forest harvesting and tree-measuring metrics and is a single-tree model for specific tree species in a specific region. The present work imitates the form of single-tree models at the plot level to find a suitable plot-level LiDAR metric to replace traditional tree-measuring metrics.

An idealized and simple tree allometry equation for special species is:

(6) $$\mathrm{AGB}={\mathrm{𝑎𝐷}}^{b}$$

Since DBH is the most easily accessible and accurately measurable tree indicator, and there is an intrinsic relationship between the DBH and tree height, the model is widely used (Chave et al., 2005).

However, Eq. 6 cannot explain the variability of diameter and tree height growth caused by tree age, forest density, site conditions and management measures; the introduction of the tree height factor is necessary.

(7) $$\mathrm{AGB}={\mathrm{𝑎𝐷}}^{b\,1}\,H^{b\,2}$$

where *H* represents the tree height (m), and *a*, *b*1, and *b*2 are fitted coefficients.

The essence of LiDAR is ranging, which can directly estimate tree height. Therefore, this paper applies Eqs 6 and 7 to Eqs 8 and 9, which are plot-averaged height-based allometry models, and fits the equations as follows:

(8) $$\mathrm{ACD}={\mathrm{𝑎𝐻}}^{b}$$

(9) $$\mathrm{ACD}=a\,{\mathrm{BA}}^{\prime ,b\,1}\,{\mathrm{TCH}}^{b\,2}\;$$

where ACD represents the aboveground carbon density (Mg C ha^–1^), and *H* represents AvgH, LorH, or BA computed from field-measured data and TCH extracted from the CHM. BA’ is the fitted result in Eq. 10.

(10) $${\mathrm{BA}}^{\prime }=a+b\,\mathrm{TCH}$$

### Percentile Model

Light detection and ranging is highly sensitive to the three-dimensional structure of forest, because laser pulses can penetrate the canopy and then record all echo signals from the ground to the canopy surface. Therefore, a series of LiDAR metrics, such as height percentile, density percentile, variation coefficient, etc., have been successively extracted to capture key information of forest canopy (Nilsson, 1996; Lefsky et al., 2002; Naesset, 2002; Nelson et al., 2004).

In this study, the height and density percentiles extracted from the NPC were used to regression fit the ACD calculated from the field investigation data. The model is as follows, and the independent variables are described in Table 1. The prediction results of this model (Eq. 11) were compared with the prediction results of the allometry models (Eqs 8, 9) proposed in this paper.

(11) lnACD=β0+β1lnh25+β2lnh50+β3lnh75+β4lnh90+β5lnh95+β6lnd25+β7lnd50+β8lnd75+β9lnd90+β10lnd95+β11lnCC+ε

### Model Fitting and Evaluation

All models in this study were fitted by the least squares (OLS) method (Meng et al., 2018). This method is simple and reliable, avoiding the algorithm differences of different fitting methods and making the fitting results more contrasting. All power models were first converted to a linear model by natural log transformation for regression fitting to correct both non-normality and heteroscedasticity. Then, the correlation of coefficients (*R*^2^) and back-log root-mean-squared errors (RMSE) were employed to compare the performance of the models, and 10-fold cross-validation analysis was used to evaluate the stability of the models.

### Model Application

**FIGURE 2:**
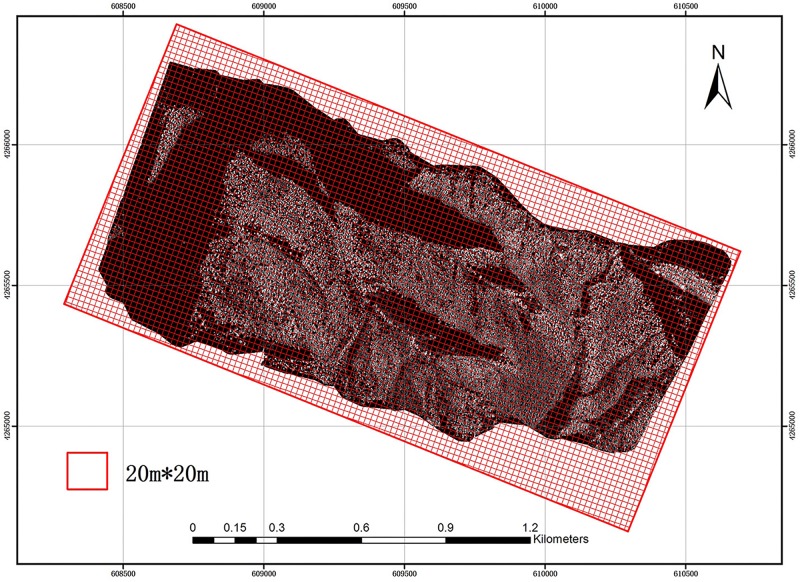
Fishnet for the study area.

The study area was divided into a 20 m × 20 m grid using GIS software (Figure 2). The size and direction of the grid were the same as those of the field plots in order to reduce possible errors. Then, LiDAR parameters were extracted using each grid from the CHM and NPC and introduced into the allometry models (Eqs 8, 11) proposed in this paper for calculation. Thereby, ACD distribution maps of the study area were obtained, compared and evaluated. Since there was no point cloud at the boundary of the study area, grid incompleteness due to cropping did not affect the final prediction.

## Results

### Field Investigation Data Analysis

**FIGURE 3:**
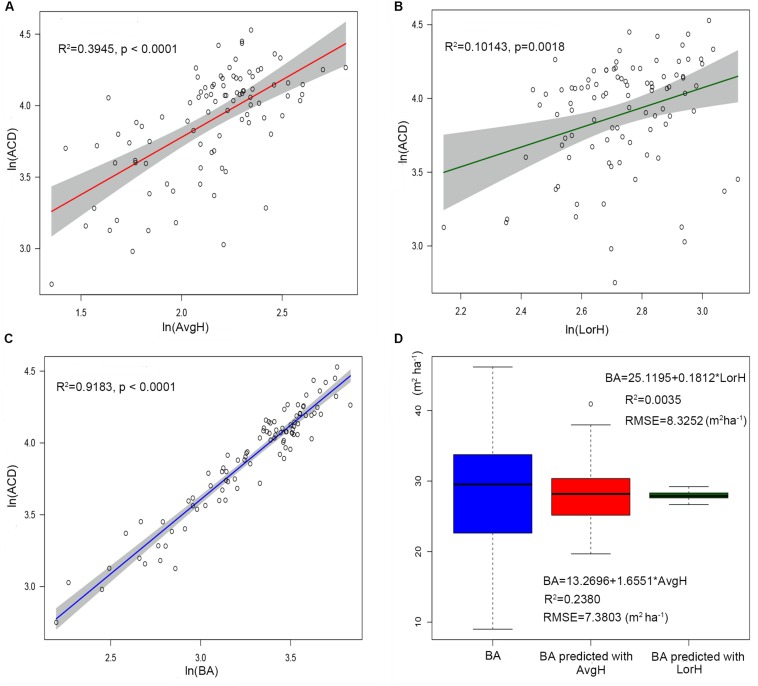
The linear relationship between ln(AvgH) and ln(ACD) **(A)**, ln(LorH) and ln(ACD) **(B)**, ln(BA)and ln(ACD) **(C)**. The shadowed region shows the 95% confidence interval. **(D)** Boxplots of the field-surveyed BA and predicted BA using plot-averaged height (AvgH) and Lorey’s height (LorH).

Using the comparison between the simple power-law model (Eq. 8) of ACD and the three plot-averaged metrics (AvgH, LorH, and BA)calculated from the field inventory, we found that the BA explains 91.8% of the variation in ACD, which is much higher than the 39.5% explained by AvgH and 10.1% explained by LorH (Table 2), and the convergence of BA is much better than that of AvgH and LorH (Figure 3), which indicates that BA is the optimal plot-averaged indicator for the inversion of ACD. It is not surprising that BA is a stronger predictor of AGB than height is, because BA can be measured with a lot better accuracy than height, and because DBH is weighted higher than tree height in Eq. 7. We also found that the exponent b is close to 1 (*b* = 1.0300), which indicates that the ACD is nearly linearly related to BA, and the cross-validated *R*^2^ value (0.9182) is reduced by 0.004 compared with the model-fitted *R*^2^ (0.9143), which indicates that the model using BA tends to be applicable and stable. This result means that, when we want to obtain the ACD of plots, we can discard the exhaustive field inventory data and only need to perform spatially explicit point-based measurements using the relascope or prism method.

**TABLE 2:** Summary for ACD estimation using AvgH, LorH, and BA.

**Model**	**Model parameters**		***R*^2^**	**Jackknifing *R*^2^**	**RMSE (Mg C ha^–1^)**
	***a***	***b***			
ACD = aAvgH^b^	8.7899	0.8026	0.3945	0.3587	13.5561
ACD = aLorH^b^	7.8821	0.6691	0.1014	0.0614	15.9938
ACD = aBA^b^	1.6528	1.0345	0.9182	0.9143	5.5440

Moreover, although AvgH and LorH are the most commonly used plot-averaged height indicators, when they were applied in Eq. 8 to predict ACD, the effect was poor, with *R*^2^ values of 0.3954 and 0.1014, respectively, and RMSE values of 13.5561 (Mg C ha^–1^) and 15.9938 (Mg C ha^–1^), respectively; the results with AvgH are slightly better than those with LorH (Table 2). This suggests that the plot-averaged height alone does not account for the variation in the ACD. Therefore, the plot-level ACD estimation (EACD) based on LiDAR should exclude the AvgH and LorH steps and directly fit ACD with LiDAR-extracted metrics (Figure 3D).

### TCH Models and the Comparisons

**FIGURE 4:**
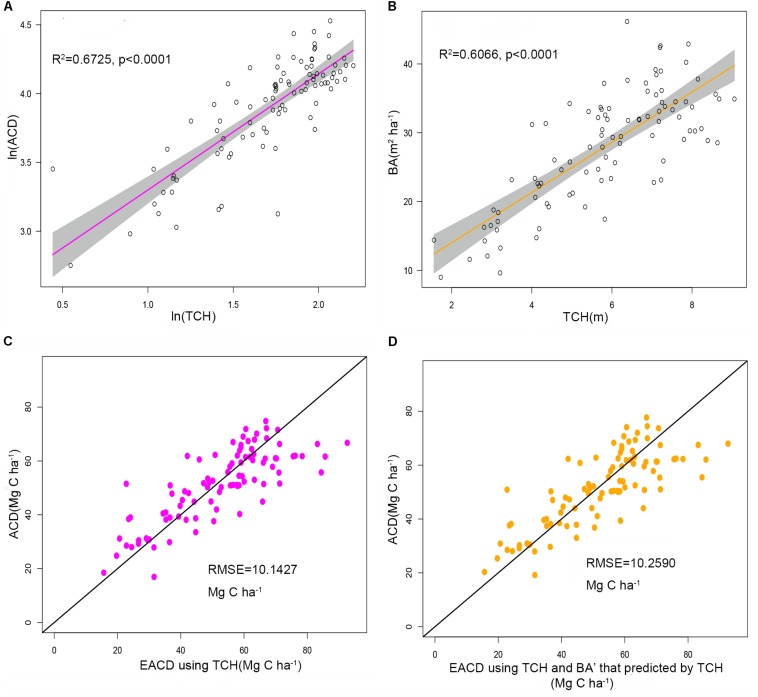
**(A)** Linear relationship between ln(TCH) and ln(ACD). **(B)** Linear relationship between TCH and the basal area (BA). The shadowed region shows the 95% confidence interval. **(C)** ACD estimated using top of canopy height (TCH) from LiDAR compared to field-surveyed ACD. **(D)** ACD estimated using TCH and BA predicted from TCH compared to field-surveyed ACD. The black line is a 1:1 reference line.

The plot-level LiDAR metric (TCH) was taken into the ideal simplest allometry (Eq. 8) and was subjected to log changes and linear fits. The result showed that TCH could explain 67.25% of the variation in ACD (Figure 4A), suggesting that TCH is a simple and effective predictor of ACD from LiDAR and that the classic allometry model (Eq. 6) can be extended from the tree-level to the plot-level scale.

Using regression by ordinary least squares, we modeled variation in BA to TCH for 94 plots, with resulting values of *R*^2^ = 0.6066 and RMSE = 5.1749 m^2^ ha^–1^ (Eq. 10, Figure 4B, and Table 3). By substituting this regression result into Eq. 9, the EACD could be generated without field inventory data. However, in a comparison of the fitting results, we found that the scatter plots were almost the same (Figures 4C,D); *R*^2^ only increased by 0.0036, and RMSE increased by 0.1163 Mg C ha^–1^ (Table 3). This strongly suggests that the daisy-chain method of TCH cannot achieve the same ACD prediction as the field-measured BA. Therefore, if we only use LiDAR-extracted TCH, the height-diameter model (Eq. 9) and height model (Eq. 8) have no essential difference in accuracy. Thus, this paper ultimately chose Eq. 8 as the final allometry model, and the parameter H used TCH.

**TABLE 3:** Summary for ACD estimation using TCH only or TCH and BA’ in pairs.

**Model**	**Model parameters**			***R*^2^**	**Jackknifing *R*^2^**	**RMSE (Mg C ha^–1^)**
	***a***	***b*1**	***b*2**			
ACD = *a*TCH*b*1	11.6592	0.8436	–	0.6725	0.6585	10.1427
BA’ = *a*+*b*1TCH	6.7117	3.6516	–	0.6066	0.5882	5.1749 (m^2^ ha^–1^)
ACD = *a*BA’*b*1TCH*b*2	0.9393	1.1977	0.0018	0.6761	0.6022	10.2590

### Percentile Model and a Comparison of Results

The following result (Eq. 12) was obtained by the multiple regression fitting of the surveyed ACD of 94 plots and the LiDAR percentile metrics listed in Table 1.

(12) lnACD=1.896+0.033⁢lnh25+12.106⁢lnd95

**FIGURE 5:**
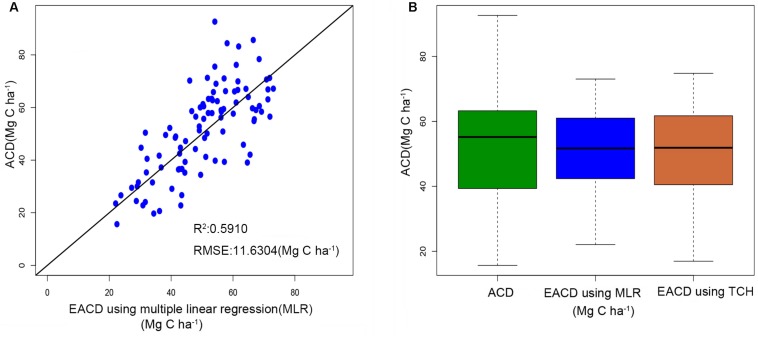
**(A)** Estimated ACD values of the percentile model versus the field investigation. **(B)** Boxplots of field-surveyed ACD and ACD estimated using the percentile model and TCH model.

The ACD in the study area is closely related to h25 and d95. These two parameters can explain 59.1% of the ACD variation, with a RMSE of 11.6304 Mg C ha^–1^ (Figure 5A), and the prediction accuracy is lower than the 67.25% of Eq. 8 using TCH. It demonstrated that the height allometry model proposed in this paper can replace the traditional LiDAR percentile model with improved precision.

Figure 5B illustrates the difference between the predicted values and the survey values of ACD. The median values of the MLR model and the TCH model are near 52 Mg C ha^–1^, which is slightly lower than the measured ACD. Furthermore, the range of predicted values of the TCH model is slightly smaller than the surveyed value range, which is larger than the range of the MLR model. Therefore, compared with the MLR model, the TCH model has a wider prediction range and can represent larger and smaller values of ACD.

### Model Application

**FIGURE 6:**
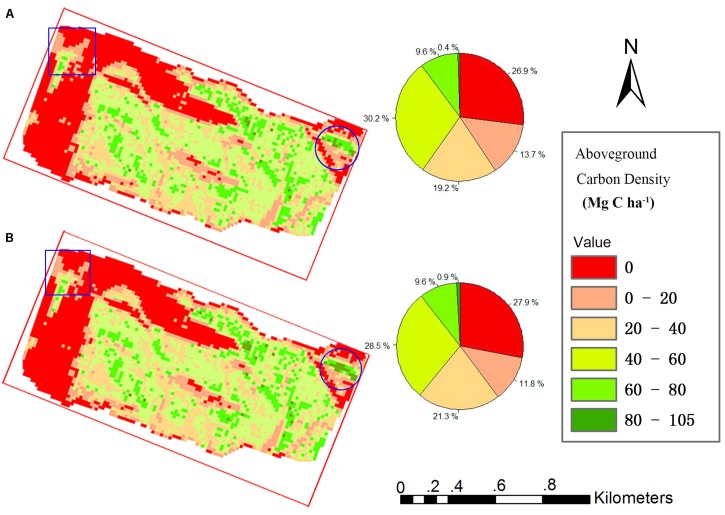
Study area carbon density map predicted using the TCH model **(A)** and percentile model **(B)**. Square is low density area, Round is high density area.

All grid values in the study area were calculated using our proposed TCH allometry model and percentile model, and then maps of ACD were produced. Figure 6 shows that the spatial distribution of the two maps is very similar. The high-density area of the map from the percentile model is slightly larger than that of the map from the TCH model (blue circle), and the low-density area demonstrates the opposite trend (blue rectangle). In addition, the density distribution percentages of the two maps are basically the same as those shown in the two pie charts. According to the grid statistics, the average ACD from the TCH model is 41.49 Mg C ha^–1^, and the maximum value is 104.70 Mg C ha^–1^, which is slightly larger than the values of 40.13 and 95.46 Mg C ha^–1^ from the percentile model. This resulted in an overall aboveground carbon reserve of the study area of 5535.54 Mg for the TCH model and 5433.06 Mg for the percentile model; the difference between the two models is only 1.89%. Although the accuracies of the TCH and the MLR models are not much different, the TCH model is much simpler and easier than the MLR model.

## Discussion

Our original purpose was to find a suitable plot-averaged LiDAR parameter and use existing allometry models to quickly and accurately predict the forest carbon density. The exponential model of TCH captures 67.25% of ACD changes (Table 3) and has a higher accuracy than the traditional percentile model of this study. We also realized that the accuracy of our prediction is relatively low. The possible reasons are (1) the plot size of 20 m × 20 m is relatively small, and the edge effect is obvious; (2) point cloud density is not enough, and the conical crown of spruce is not captured accurately and (3) the penetration rate of point cloud is insufficient, and the detection of lower wood is limited. However, for the research objective, we did effectively improve the accuracy of LiDAR’s prediction of ACD, simplify the prediction steps and solidify the form of the prediction model.

**FIGURE 7:**
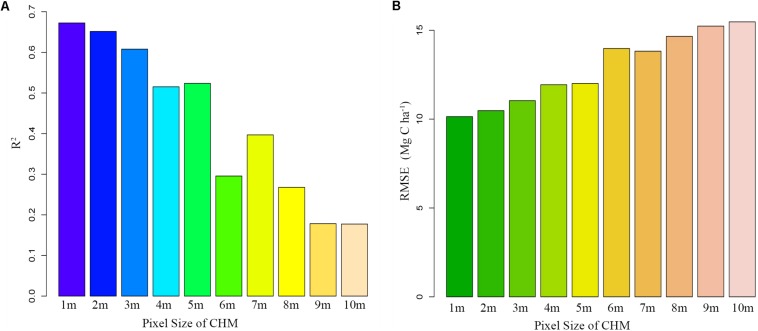
Fitting trends of TCH and ACD under different CHM precisions. **(A)** Declining *R*^2^; **(B)** Increasing RMSE.

Moreover, since the TCH is derived from the mean of the CHM based on the plots, the TCH is also subject to the pixel size. We extracted 10 CHMs from the LiDAR point cloud, with pixel sizes from 1 to 10 m, and then extracted the corresponding TCHs to fit the ACD. As the pixel size increased, *R*^2^ continually decreased, and the RMSE continually increased (Figure 7). This result indicated that the smaller the CHM pixel is, the better the fitting effect of TCH will be. This study was limited to a point cloud density of 3.43/m^2^, so the minimum pixel size was 1 m. In addition, we found that when the pixel sizes were 5 and 7 m, the fitting effect fluctuated slightly, but this fluctuation did not affect the overall law. The reason for this finding requires further study. Similarly, fitting accuracy is also limited by the size of the plot and the number of samples. A larger plot size means a smaller boundary effect, and a larger plot number means a smaller outlier influence (Ni et al., 2014; Gwenzi and Lefsky, 2017). However, larger plots are more expensive and time-consuming than smaller plots, so finding an optimal plot size and number in coniferous forest will be an important task for future study.

**FIGURE 8:**
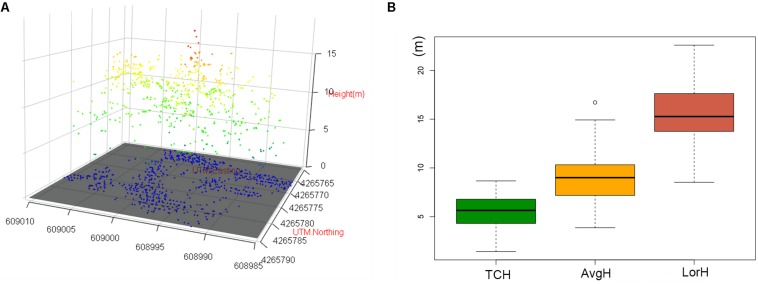
**(A)** 3D scatter plot of the normalized point cloud (NPC) in plot 1. **(B)** Boxplots of TCH, AvgH and LorH. White bullet is mild outliers of boxplot.

The three-dimensional visualization of the point cloud in plot no. 1 (Figure 8A) suggests that the forest point cloud includes the crown, some of the lower layer, some of the trunks and the woodland gap. Therefore, the TCH data derived from the point cloud also contain the above information. However, the average height of the plot (AvgH) ignores the forest gap and is therefore slightly higher than the TCH (Figure 8B). Although LorH is widely used for the estimation of forest biomass (Mitchard et al., 2012; Gwenzi and Lefsky, 2016), LorH is mainly used to evaluate site quality and mostly reflects the largest trees in the forest; therefore, its value is larger than that of TCH and AvgH (Figure 8B), and LorH is not applicable for fitting the ACD of irregular and mixed forests. This explains why TCH is the optimal ACD predictor.

We also recognized a flaw in the ACD prediction at the plot scale. Whether in the field measurement phase, the plot-based TCH extraction phase, or the final ACD prediction phase, our resolution is fixed at 20 m × 20 m. This inevitably leads to the conversion of the continuous ACD distribution in nature into a discontinuous distribution, which may cause a large jump phenomenon at the boundary. Therefore, selecting the appropriate interpolation algorithm to restore the continuity of the ACD will help improve the prediction accuracy of our proposed models (Loquin and Strauss, 2010; DeWitt et al., 2017). In addition, we only adopted linear regression fitting based on the least squares method in this paper, and although this method is simple and practical, it is not necessarily the best method. With the rise of machine learning in LiDAR research (Zhou et al., 2017; Jin et al., 2018; Lin et al., 2018), it will be necessary to compare various machine learning algorithms in future research to find the best way to fit the allometry models.

Finally, we must emphasize that although our proposed TCH-based allometric approach is an efficient LiDAR-assisted ACD prediction method, the allometry model used for plot calculation is generally targeted to a specific region and species (Picard et al., 2015; Duque et al., 2017), so it is necessary to re-select an appropriate allometry model for other tree species and ecological regions when our method is used. Moreover, developing a general ACD prediction model based on LiDAR for forests across ecological regions and species will be the focus of our future research.

## Conclusion

Using the traditional allometry growth model theory, this paper proposed two models based on TCH extracted from LiDAR data. The first model was a simple power model (only using TCH) based on the diameter allometry, and the second model was a daisy-chain model (TCH → BA′ → ACD) based on diameter-height allometry. A comparison of the results suggested there was little difference in the fitting accuracy and error distribution between models. In addition, this paper compared the traditional LiDAR percentile method with the proposed method and found that the latter method had a higher precision, fewer parameters, more concise steps and more stable forms than the former method. Furthermore, the implicit hypothesis in our study, the traditional allometry model of individual trees can be extrapolated to the plot scale, was confirmed. The LiDAR-assisted ACD estimation method proposed in this study will accelerate the application of airborne LiDAR technology in forest carbon density measurements and provide an accurate data basis for forest ecosystem research.

## Data Availability

Publicly available datasets were analyzed in this study. This data can be found here: http://www.heihedata.org/data/.

## Author Contributions

HH and WL: conceptualization, methodology, and writing-original draft preparation. HH: software, resources, and data curation. XZ and PZ: validation. HH and XZ: formal analysis. HH, WL, and XZ: investigation. QC: writing, review, and editing. HH and PZ: visualization. QC and PZ: supervision, project administration, and funding acquisition.

## Conflict of Interest Statement

The authors declare that the research was conducted in the absence of any commercial or financial relationships that could be construed as a potential conflict of interest.

## Footnotes

1 http://www.lastools.org
